# Unifying Endotracheal Intracuff-Pressure Monitoring in the Intensive Care Unit: Bridging the Gap Between Standardization and Neglect

**DOI:** 10.7759/cureus.56875

**Published:** 2024-03-25

**Authors:** Sankalp Yadav, Gautam Rawal

**Affiliations:** 1 Medicine, Shri Madan Lal Khurana Chest Clinic, New Delhi, IND; 2 Respiratory Medical Critical Care, Max Super Speciality Hospital, New Delhi, IND

**Keywords:** intracuff pressure, mechanical ventilation (mv), extubation, tracheomalacia, ventilator-associated pneumonia (vap), endotracheal tube

## Abstract

Endotracheal cuff-pressure monitoring is a critical component of patient care in the intensive care unit, ensuring the safety and efficacy of mechanical ventilation. Despite its importance, there remains a lack of standardized protocols regarding optimal pressure targets and documentation practices. This editorial examines the significance of endotracheal intracuff-pressure monitoring in enhancing patient outcomes, highlighting the challenges and potential solutions in clinical practice.

## Editorial

In the busy field of critical care medicine, meticulous attention to detail is paramount. The endotracheal tube (ETT) serves a fundamental role in safeguarding a patient's airway from the risk of aspiration of gastric contents and facilitating positive pressure ventilation in various medical settings, including anesthesia, the post-anesthesia care unit, and the intensive care unit (ICU). Over time, the development, evolution, and modification of the ETT have significantly contributed to minimizing aspiration risks, aiding in lung isolation, ensuring a clear surgical field during general anesthesia, monitoring laryngeal nerve function during surgical procedures, preventing airway fires during laser surgery, and facilitating medication administration. Modern ETTs predominantly utilize polyvinyl chloride material and feature a high-volume, low-pressure cuffed design that conforms optimally to the tracheal anatomy. The cuff, located near the distal end of the ETT, is typically inflated with air to establish an effective and airtight seal [1].

Patients admitted to the ICU often require mechanical ventilation to sustain life, making the ETT a vital conduit for delivering oxygen and facilitating ventilation. However, despite its importance, the ETT can also pose risks, particularly if not managed appropriately. Endotracheal intracuff-pressure management is a crucial aspect of ensuring patient safety and optimizing outcomes in the ICU setting [1]. In this editorial, we touch on the significance of endotracheal intracuff-pressure monitoring, its clinical implications, challenges, and potential solutions, with a focus on enhancing patient care and safety.

In the medical community, there is quite a bit of variability when it comes to figuring out the perfect pressure targets and documenting them properly, despite the general understanding that measuring and monitoring intracuff pressure is crucial. Most healthcare providers tend to stick with cuff pressures ranging between 20 and 30 cmH2O. It is strongly recommended to directly measure intracuff pressure whenever possible [1]. Also, it is important to take into consideration factors such as patient specifics, the type of cuff being used, the initial pressure set, the measuring device used, and even the size of the ETT [2]. These little details can all impact intracuff pressure and need to be factored in for optimal patient care.

Importance of endotracheal intracuff-pressure monitoring

The endotracheal intracuff serves several essential functions, including preventing aspiration of oropharyngeal secretions, maintaining positive pressure during mechanical ventilation, and minimizing the risk of ventilator-associated complications such as aspiration pneumonia and ventilator-associated pneumonia (VAP). However, maintaining an appropriate intracuff pressure is critical to achieving these goals effectively. Insufficient intracuff pressure can lead to microaspiration of secretions, potentially causing pneumonia, while excessive pressure may result in tracheal mucosal damage, ischemia, and pressure necrosis. Thus, precise monitoring and maintenance of intracuff pressure within optimal ranges are paramount to mitigating these risks and enhancing patient outcomes [3].

Clinical implications

Endotracheal intracuff-pressure monitoring is integral to preventing complications associated with improper cuff inflation. Studies have shown that routine intracuff-pressure monitoring significantly reduces the incidence of VAP and other ventilator-associated complications, which could affect the length of ICU stay and improve patient outcomes. However, these had limitations [4]. Furthermore, maintaining appropriate intracuff pressure is particularly crucial for patients at higher risk, such as those with altered levels of consciousness, compromised airway reflexes, or prolonged mechanical ventilation. Nevertheless, several complications can occur due to inappropriate intracuff pressure (Table 1) [1,5].

**Table 1:** Complications due to inappropriate intracuff pressure [1,5] ETT, endotracheal tube

Effects of low intracuff pressure than optimum in ETT	Effects of higher intracuff pressure than optimum in ETT
Aspiration pneumonitis and pneumonia/ventilator-associated pneumonia	Ischemia of the tracheal mucosa progressing to inflammation, ulceration, or severe ischemia, causing damage to the wall
Bronchitis	Tracheal rupture
Accidental extubation	Laryngeal stenosis
Self-extubation	Post-extubation stridor
	Subglottic stenosis due to granulation
	Trachea-carotid artery erosion
	Trachea esophageal fistula
	Scarring
	Tracheomalacia
	Damage to the recurrent laryngeal nerve
	Hoarseness
	Herniation of the cuff balloon

Challenges and solutions

Despite its importance, effective ETT cuff-pressure monitoring faces several challenges in clinical practice. One of the primary challenges is the lack of standardized protocols for cuff-pressure measurement, frequency of measurement, and management across different healthcare settings. Variability in equipment, techniques, and staff training can contribute to inconsistencies in cuff-pressure management practices. Moreover, the dynamic nature of ETT cuff pressures, influenced by factors such as patient movement, changes in ventilator settings, and variations in airway anatomy, poses additional challenges to maintaining optimal intracuff-pressure levels [1,5]. Besides, the various ways of measuring the cuff pressures are given in Table 2 [1,5].

**Table 2:** Techniques for measuring ETT intracuff pressure ETT, endotracheal tube

Techniques for measuring ETT intracuff pressure
Manual palpation of the pilot balloon
Minimum leak technique
Minimum occlusive volume
Predetermined volume technique
Analogue/digital manometer
Pressure-sensing syringe for ETT cuffs
Mobile terminal application program
Direct intracuff pressure monitoring
Automatic control devices
Air bubble technique

The potential of automatic pressure control devices is encouraging, yet their efficacy in maintaining optimal cuff pressure ranges varies across different models. Furthermore, their impact on preventing VAP also demonstrates discrepancies among devices, underscoring the necessity for additional research and validation in this domain [5]. To address these challenges, multidisciplinary collaboration and standardized protocols are essential. Education and training programs for healthcare providers should emphasize the importance of regular cuff-pressure monitoring and provide guidelines for appropriate cuff inflation techniques. Additionally, the use of innovative technologies, such as automated cuff-pressure monitoring devices, can streamline the monitoring process and facilitate real-time adjustments to maintain optimal cuff pressures. These technologies offer the potential to improve the accuracy and reliability of cuff-pressure management while reducing the burden on healthcare staff.

Future directions

Looking ahead, further research is needed to refine ETT intracuff-pressure monitoring strategies and optimize patient outcomes. Prospective studies evaluating the efficacy of automated cuff-pressure monitoring devices compared to traditional methods are warranted to establish their superiority in clinical practice. Additionally, investigating the impact of cuff-pressure management protocols on long-term outcomes, such as mortality and morbidity, will provide valuable insights into the broader implications of this intervention.

ETT intracuff-pressure monitoring plays a pivotal role in ensuring the safety and efficacy of mechanical ventilation in the ICU. By preventing complications associated with improper cuff inflation, such as VAP and tracheal injury, meticulous cuff-pressure management contributes to improved patient outcomes and enhanced quality of care. Addressing the challenges associated with intracuff-pressure monitoring through standardized protocols, education, and technological advancements is crucial for maximizing its clinical impact. As we strive for excellence in critical care medicine, prioritizing endotracheal intracuff-pressure monitoring underscores our commitment to patient safety and well-being in the ICU setting.
